# Anterior cruciate ligament (ACL) reconstruction with quadriceps tendon autograft and press-fit fixation using an anteromedial portal technique

**DOI:** 10.1186/1471-2474-13-161

**Published:** 2012-08-27

**Authors:** Ralph Akoto, Juergen Hoeher

**Affiliations:** 1 Department of Trauma and Orthopedic Surgery, University of Witten/Herdecke, Cologne Merheim Medical Center, Ostmerheimer Straße 200, Cologne, 51109, Germany; 2 Clinic for Sports Traumatology at Cologne Merheim Medical Center, Ostmerheimer Staße 200, Cologne, 51109, Germany

## Abstract

**Background:**

This article describes an arthroscopic anterior cruciate ligament (ACL) reconstruction technique with a quadriceps tendon autograft using an anteromedial portal technique.

**Methods:**

A 5 cm quadriceps tendon graft is harvested with an adjacent 2 cm bone block. The femoral tunnel is created through a low anteromedial portal in its anatomical position. The tibial tunnel is created with a hollow burr, thus acquiring a free cylindrical bone block. The graft is then passed through the tibial tunnel and the bone block, customized at its tip, is tapped into the femoral tunnel through the anteromedial portal to provide press-fit fixation. The graft is tensioned distally and sutures are tied over a bone bridge at the distal end of the tibial tunnel. From the cylindrical bone block harvested from the tibia the proximal end is customized and gently tapped next to the graft tissue into the tibial tunnel to assure press fitting of the graft in the tibial tunnel. The distal part of the tibial tunnel is filled up with the remaining bone.

All patients were observed in a prospective fashion with subjective and objective evaluation after 6 weeks, 6 and 12 months.

**Results:**

Thirty patients have been evaluated at a 12 months follow-up. The technique achieved in 96.7% normal or nearly normal results for the objective IKDC. The mean subjective IKDC score was 86.1 ± 15.8. In 96.7% the Tegner score was the same as before injury or decreased one category. A negative or 1+ Lachman test was achieved in all cases. Pivot-shift test was negative or (+) glide in 86.7%. The mean side-to-side difference elevated by instrumental laxity measurement was 1.6 ± 1.1 mm. Full ROM has been achieved in 92.3%. The mean single one-leg-hop index was 91.9 ± 8.0 at the follow-up.

**Conclusions:**

Potential advantages include minimum bone loss specifically on the femoral side and graft fixation without implants.

## Background

To date several techniques for anterior cruciate ligament (ACL) reconstruction have been published. Quadriceps tendon grafts are currently considered only a second choice graft, although clinical studies have demonstrated good results and a low donor site morbidity [1-5]. However, surgical techniques including graft fixation for quadriceps tendon vary widely. More recently, several authors have favoured press-fit fixation of the graft with a more biological approach avoiding screw fixation and the use of implants. To date all press fit techniques for ACL reconstruction using a quadriceps tendon graft have used a transtibial single incision technique [1-4,6]. However, several studies have demonstrated that the transtibial approach may result in a non-anatomic placement of the femoral bone tunnel [7,8]. Therefore, it was the goal of this study to develop a surgical technique for ACL reconstruction in which the advantages of using a quadriceps graft and the advantage of using a press fit technique for fixation were achieved when the femoral tunnel was drilled through an anteromedial portal.

## Methods

### Surgical technique

After routine arthroscopy including meniscus and cartilage surgery a low anteromedial portal was placed 1 cm medial to the patellar tendon and close to the superior tibial edge with the knee flexed to 90°. This portal was slightly enlarged to be used as the “working” anteromedial portal [9].

### Preparation of ACL insertions

When complete ACL rupture has been verified intercondylar notch preparation was performed until anatomical landmarks, such as the medial aspect of the PCL and the over-the-top position could by identified. On the tibial side the infrapatellar fat pad was partially resected until the anterior horn of the lateral meniscus can be clearly visualized. The tibial footprint was cut longitudinally preserving its proprioceptive and vascular contributions so that the graft would later be surrounded by the remnants of the tibial stump.

### Graft harvest

**Figure 1:**
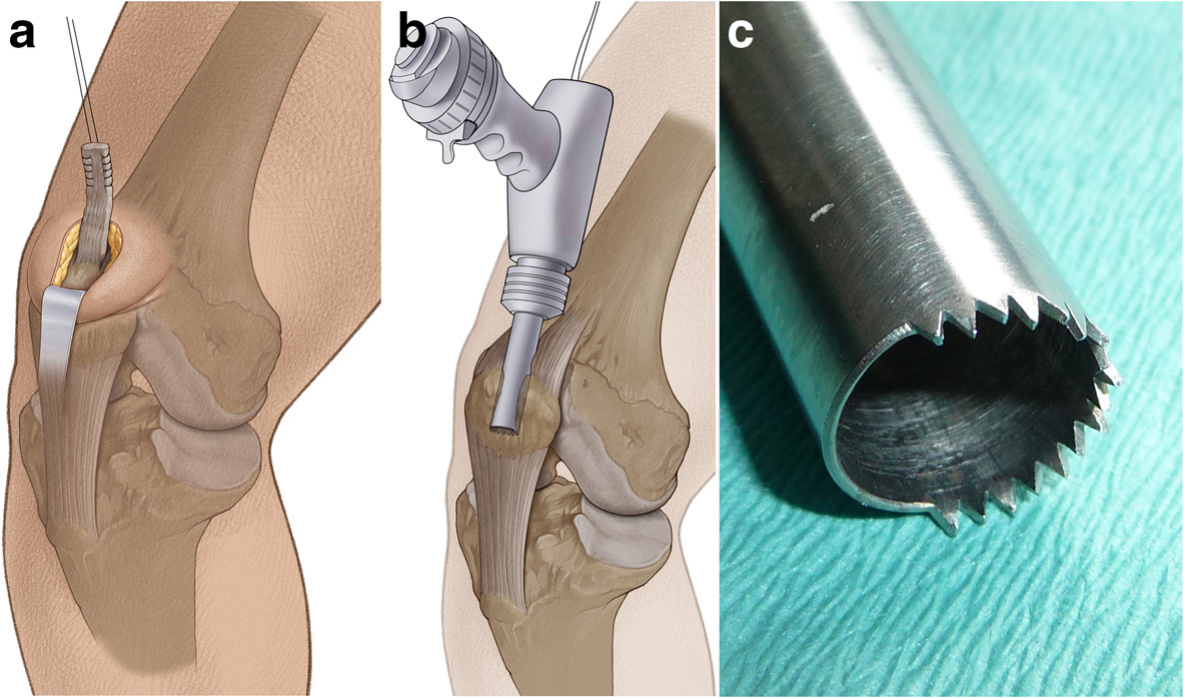
**Harvesting the quadriceps tendon graft. a**. The proximal end of graft whipstitched using # 2 polyester suture. **b**. The sutured end of the graft was threaded through a hollow burr, which was attached to an oscillating compression air drill (Richard Wolf, Knittlingen, Germany). **c**. The cutting edge of a 9.4 mm hollow burr for graft harvesting. Two thirds of the circumference of this specific hollow burr had sharp teeth, while the other 1/3 was blunt. Using the hollow burr with in inner diameter of 9.4 mm, a cylindrical bone cylinder of 20 mm length could be harvested from the proximal aspect of the patella.

We used a 4–5 cm longitudinal incision starting at the proximal aspect of the patella running proximally and centred over the quadriceps tendon. A 10 mm wide and 50 mm long strip of tendon was excised. Care was taken to harvest all layers of the quadriceps tendon, leaving the suprapatellar pouch of the joint intact. The proximal end of the graft was “whipstitched” using a #2 Ultrabraid® suture (Smith & Nephew, Inc.) (Figure 1a).

**Figure 2:**
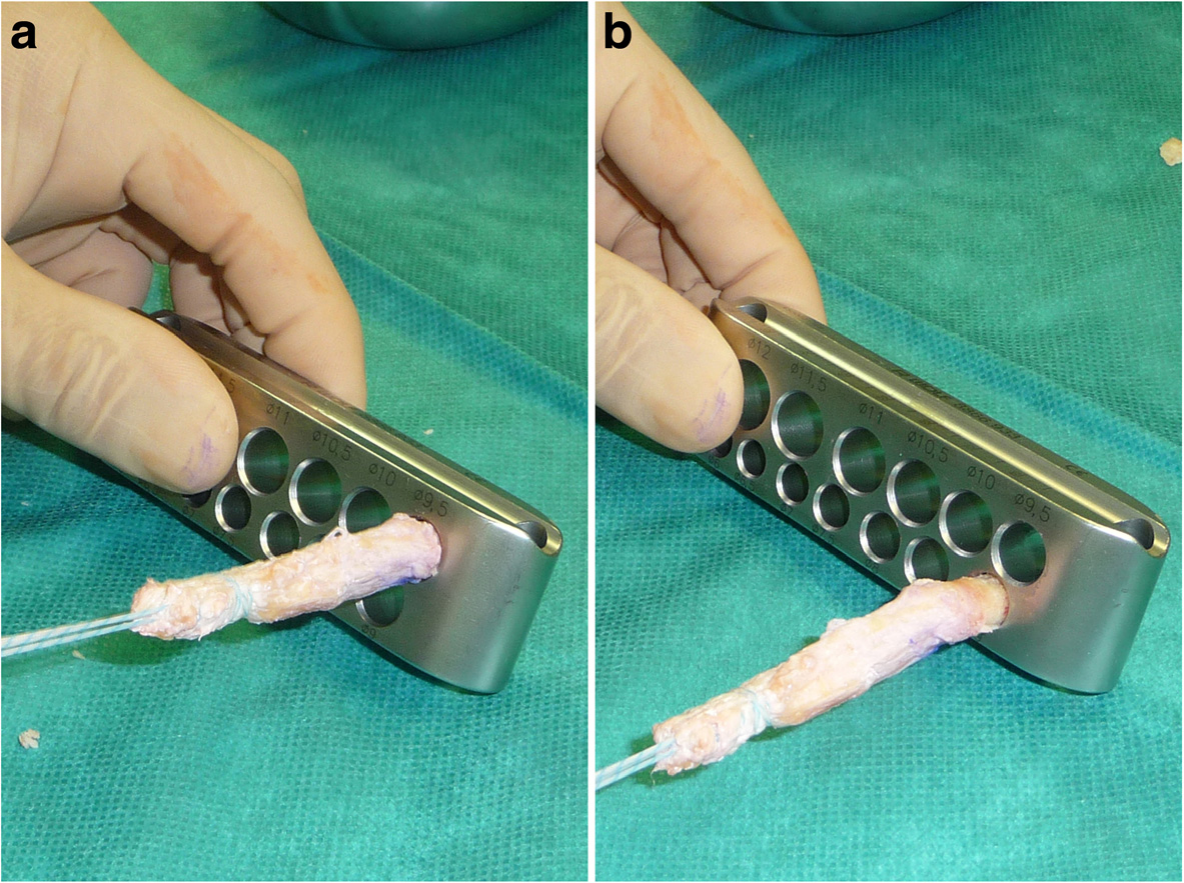
**The cylindrical 20 mm bone block at the end of the graft had a diameter of 9.4 mm and a graft sizer was used to ensure that it could be completely passed through a 9.4 mm template.b**. For later easier graft placement in the femoral tunnel, the apex of the bone block was prepareted in a fashion, that its distal 1 cm just fitted into a 9.0 mm template.

The sutured end of the graft was then threaded through a hollow burr, which was attached to an oscillating compression air drill (Richard Wolf, Knittlingen, Germany). Two thirds of the circumference of this specific hollow burr had sharp teeth, while the other 1/3 was blunt (Figure 1b, c). Using the hollow burr with an inner diameter of 9.4 mm, a cylindrical bone cylinder of 20 mm length could be harvested from the proximal aspect of the patella in the technique described by Boszotta [3]. Thus, a graft with a strip of 50 mm quadriceps tendon attached to a cylindrical bone block 20 mm in length was available to be used for ACL reconstruction. The cylindrical shaped bone block had a diameter of 9.4 mm and a graft sizer was used to ensure that it could be completely passed through a 9.4 mm template (Figure 2a). The tendon defect was closed with a running suture.

**Figure 3:**
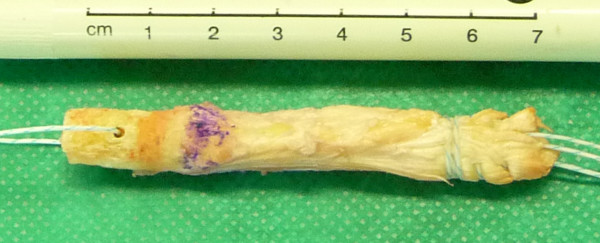
Complete graft with a 50 mm strip of quadriceps tendon and a 20 mm cylindrical bone plug.

On the back table the bone block was then tapered with the ranguer in a fashion that its distal 1 cm just fits into a 9.0 mm template (Figure 2b). Then, a 1.6 mm hole was drilled through the bone block and a polyester suture was introduced for later graft passage. Finally, a second #2 Ultrabraid® suture (Smith & Nephew, Inc.) was whipstitched to the proximal tendon end (Figure 3).

### Tunnel placement and preparation

**Figure 4:**
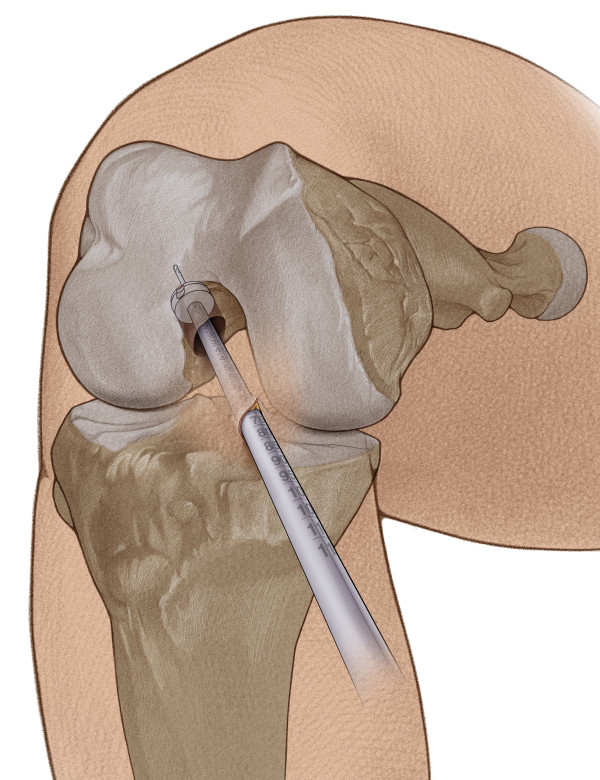
**The right knee was flexed to 120. **After a 2.4 mm guide wire was introduced into the lateral femoral condyle through the anteromedial portal, an 8.0 mm cannulated reamer was used to create a socket into the femur of 20 mm in length.

At 90° knee flexion, a femoral drill guide with a 7 mm offset hook was introduced through the anteromedial portal. The offset hook was placed in the over the top position and the knee is gently bent to 120° of flexion. After introduction of a 2.4 mm guide wire into the lateral femoral condyle through the drill guide an 8.0 mm cannulated reamer was used to create a socket into the femur of 20 mm in length. The femoral bone tunnel was further impacted with a 9.0 mm compactor to a depth of approximately 22 mm (Figure 4).

For creating the tibial tunnel, a tibial drill guide (device “Heidelberg” Richard Wolf, Knittlingen, Germany) was introduced through the anteromedial portal and placed with the alignment hook into the centre of the tibial footprint of the ACL. A 2–3 cm longitudinal skin incision was made on the medial aspect of the tibia so that the drill guide could be placed on the anteromedial aspect of the tibia. A 2.4 mm guide wire was then introduced into the joint through the drill guide. Then the guide wire sleeve was replaced by a drill sleeve, so that a 10.5 mm hollow burr could be used for creating the tibial tunnel. Thus, a cylindrical bone block could be retrieved from the tibial bone.

A cortical bone bridge of about 1 cm length was created distal to the exit of the tibial tunnel and a suture loop was passed around it for later graft fixation.

### Preparation of the bone plug retrieved from the tibia

On the back table the bone cylinder retrieved from the tibia was divided into three parts. Additionally, the proximal bone plug of about two cm in length was longitudinally split so that it could be used as a wedge for compressing the graft tissue in the bone tunnel. The middle part of the bone plug might be used to fill up the bony defect in the patella, while the distal part was used to fill up the tibial tunnel distally at the end of surgery.

### Graft placement and graft fixation

With the bone block running first the graft was then introduced through the tibial tunnel into the joint in a common fashion. Thus the bone block could easily slide into the femoral bone tunnel to half of its length.

**Figure 5:**
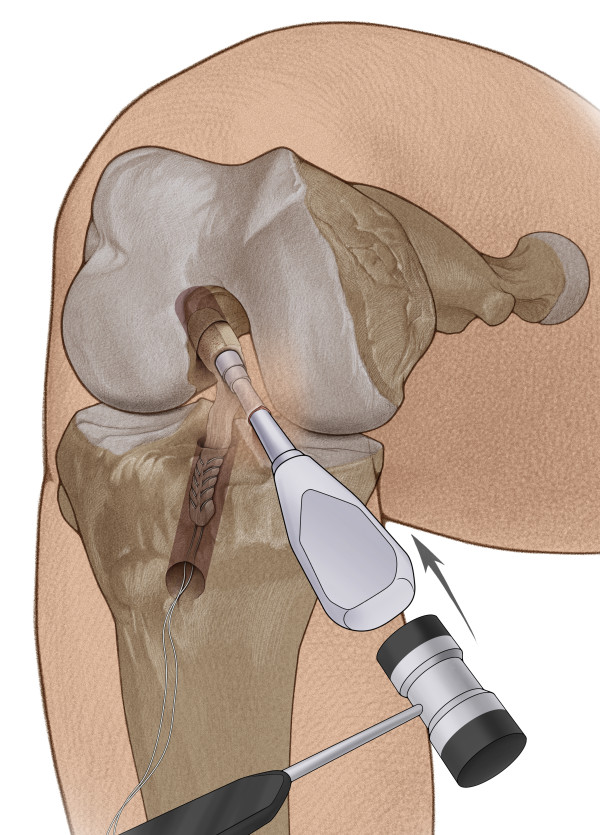
**With the knee flexed to 120°, the bone cylinder was pulled into the femoral tunnel. **Then an impactor was used to tap the bone cylinder into the femoral tunnel until the bone lock is flush with the femoral cortex.

**Figure 6:**
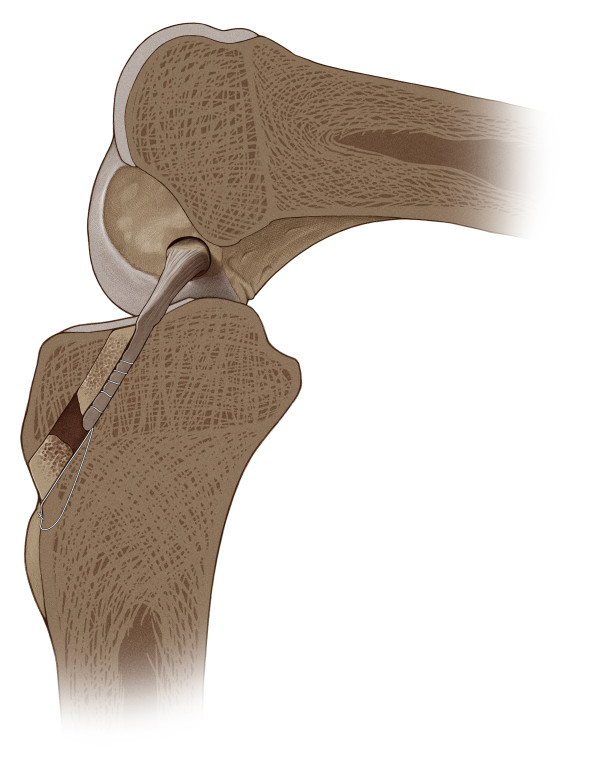
**The bone block of the graft was flush with the femoral cortex with press-fit fixation. **Tibially, a split bone wedge harvested from the tibia the graft was compressed against the bone tunnel wall close to the joint line, furthermore the graft was secured with sutures tied over a bone bridge. The remaining part of the harvested bone plug was stuffed into the tibial tunnel like a cork to close the bone tunnel distally.

Holding tension on the femoral sutures and keeping the knee flexed to 120°, a straight impactor was now introduced into the joint through the anteromedial portal. Under arthroscopic control the bone block could be now gently be tapped into the femoral tunnel until the bone block lied flush with the entrance of the femoral bone tunnel (Figure 5). Bringing the knee towards extension the graft was pulled distally with the sutures exiting the tibial tunnel to ensure press-fit fixation and absolute stability of the bone block on the femoral side. After moving the knee through the range of motion half of the exiting sutures distally were passed through the additional cortical hole so that sutures could be sequentially be tied over the bone bridge at about 30° of knee flexion. Then the shimmed, split bone plug could be introduced onto the anterior aspect of the tendon tissue in the tibial tunnel. Now it could gently be tapped proximally alongside the graft tissue up to the joint line, thus providing compression of the graft against the tibial bone tunnel wall. The distal bone plug retrieved from the tibia was used to fill up the distal part of the tibial tunnel (Figure 6). Any remaining bone material was used to fill up the bony defect in the patella. Subsequentially the wounds were closed in a common fashion.

All patients followed a standardized rehabilitation protocol with partial-weight bearing and limitation of active and passive ROM to 0/0/90 in a hinged knee brace for three weeks. Return to sports was enabled after six to nine month if muscle strength is greater than 90° of the contralateral side (Table 1).

**Table 1:** Rehabilitation protocol


day 0-5	· Immobilization in a 0° splint
	**·** Lymphatic drain, cryotherapy
	**·** Three point gait, partial-weight bearing (up to 15 kg)
	**·** Active/passive range of motion 0/0/60
day 5 -21	**·** Active/passive range of motion 0/0/90
	**·** Three point gait, partial-weight bearing (up to 20–30 kg) in a brace
	**·** Lymphatic drainage, cryotherapy
	**·** Quadriceps strengthening
day 21-42	**·** Full weight-bearing without brace
	**·** Full active/passive range of motion
	**·** Quadriceps strengthening
	**·** Cycling, ergometer
week 6-12	**·** Muscle-strength training
	**·** Swimming, aquajogging
week 13-26	**·** Continuing strengthening exercises
	**·** Running if tolerated
6-9 months	**·** Return to sports if muscle strength greater than 90° of the contralateral side

**Figure 7:**
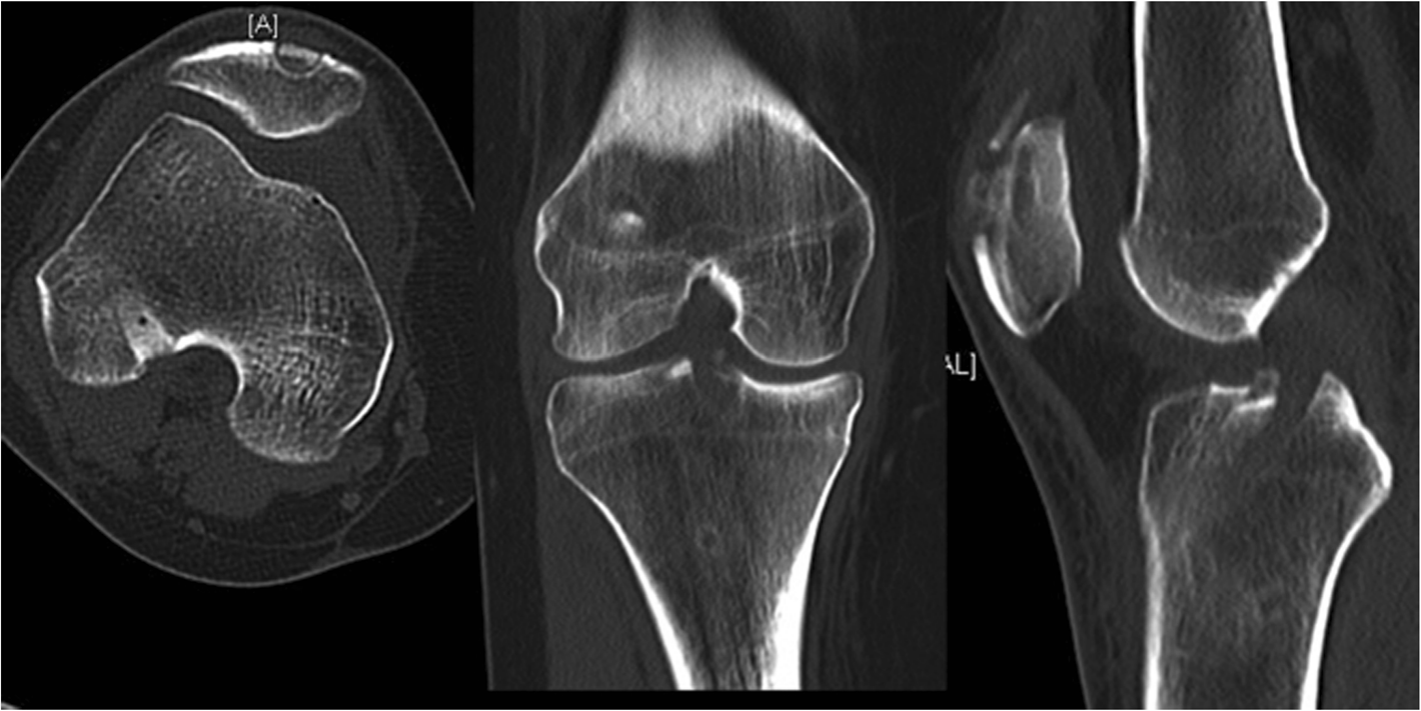
CT scan of the right knee 6 weeks postoperatively.

A CT scan six weeks after surgery was obtained to evaluate amount of bone loss and the tunnel healing quality (Figure 7).

### Inclusion and exclusion criteria of patients

In our institute active patients with anterior cruciate ligament rupture participating in pivoting or high valgus stress sports, such as judo, wrestling, or soccer, as well as patients with concomitant medial collateral ligament (MCL) insufficiency were preferably treated with a quadriceps tendon graft in the described technique. The indication for this graft selection in this population was to save hamstring function, specifically for medial dynamic stabilization of the knee.

Since 2010, all patients, who underwent ACL reconstruction using either hamstring or quadriceps tendon graft were enrolled in a prospective data base analysis and defined follow-up evaluations after approval before the beginning of treatment.

Patients with a history of previous ACL reconstruction, concomitant PCL injuries as well as reconstructive cartilage repair other than microfracturing were excluded from the study.

All patients were assessed before surgery, at 6 weeks, 6 and 12 months after surgery, using the International Knee Documentation Committee (IKDC) and Tegner score [10]. Laxity was tested with the Lachman test (graded negative, 1+, 2+ and 3+) and the pivot-shift test (graded negative, (+) glide, + and ++). Instrumental laxity measurement was performed by using the Rolimeter (Aircast Europa GmbH, Neubeuern, Germany), the knee in 20° of flexion, putting a standardized knee roll under the thigh and using maximal force, compared to the contralateral side. Knee function was assessed with ROM (lack of extension: < 3% = none, 3 to 5% moderate, 6 to 10% mild, > 10% serve; lack of flexion: < 5% none, 6 to 15% moderate, 16 to 25% mild and > 25% serve) and one leg hop index (distance hopped on the involved leg divided by the distance hopped on the noninvolved leg x 100) after 12 months. Patellofemoral crepitation elicited by extension against slight resistance was graded in crepitation with none, moderate, mild, severe pain.

## Results

Since 2010 eighty-seven patients (75 male, 13 female) underwent ACL reconstruction with the described technique. Their mean age at operation was 31 (range 16–47) years. Secure graft fixation using the press-fit technique was achieved in all cases. There were no other intraoperative complications. Postoperatively no major complications (infection, deep vein thrombosis) were observed. Within this group there was no case of rerupture or persistent effusion.

Thirty patients have been evaluated at a 12 months follow-up. The averaged results at follow-up were summarized in Table 2. The mean operation time was 102.1 ± 12.0 min. In 96.7% normal or nearly normal results for the objective IKDC were achieved, sixteen patients (53.3%) graded normal, thirteen (43.3%) nearly normal and one (3.3%) abnormal. The mean subjective IKDC score was 86.1 ± 15.8.

**Table 2:** Follow-up results one year after surgery (n = 30)


Operation time [min] (m + −std)	102.1 ± 12.0
Single one-leg-hop index (m + −std)	91.9 ± 8.0
Instrumented laxity measurement [mm] (m + −std)	1.6 ± 1.1
IKDC subjective score (m + −std)	86.1 ± 15.8
IKDC objective (normal/nearly normal)	29 (96.7%)
ROM full extension (< 3° deficit)	29 (96.7%)
ROM full flexion (> 5 ° deficit)	29 (96.7%)
Lachman test (neg. or 1+)	30 (100%)
Pivot-shift test (neg. or (+) glide)	26 (86.7%)
Patellofemoral crepitation (moderate or mild)	3 (10%)
Change in Tegner score at follow-up ≤ 1	29 (96.7%)

Twenty-six patients (86.7%) achieved the same Tegner score as before injury, three (10%) decreased one category, one (3.3%) decreased two categories.

Full ROM has been achieved in 92.3%, one patient had a mild lack of extension and one had a mild lack of extension and flexion. The mean single one-leg-hop index was 91.9 ± 8.0. The Lachman test was graded negative in 27 cases (90%) and 1+ in three cases (10%). Pivot-shift test was graded 25 times negative (83.3%), one time (+) glide (3.3%) and four times + (13.3%). The mean side-to-side difference elevated by instrumental laxity measurement was 1.6 ± 1.1 mm. Mild or moderate patellofemoral crepitation was observed in 10% at the follow-up, two patients moderate and one mild (Table 2).

## Discussion

Quadriceps tendon grafts have been shown to provide good strength and low donor site morbidity, therefore being an alternative to hamstring grafts even in primary ACL reconstruction [5,11,12]. With respect to surgical technique several authors have emphasized the need for placing the graft in an anatomical position on the femoral side. Therefore an anteromedial portal technique for femoral drilling has been advocated, because the femoral footprint can more easily be reached compared to a transtibial drilling technique [7]. Press-fit fixation techniques for an ACL graft have been suggested by several authors with various grafts (Table 3). For the quadriceps tendon graft, press fit fixation has been described only with a transtibial approach [1]. With the technique described in this paper, drilling of the femoral bone tunnel and press-fit fixation of the quadriceps tendon graft was achieved through an anteromedial portal. Femoral fixation of the bone block can even be easier performed through the anteromedial portal compared to the transtibial technique, since it can be introduced in a straight direction with a straight impactor.

**Table 3:** Surgical techniques for ACL reconstruction using press-fit fixation

**Author**	**Graft**	**Technique**	**Journal**	**Year**
Boszotta [3]	patellar tendon	transtibial	Arthroscopy	1997
Paessler [2]	hamstrings;patellar tendon	anteromedial	Orthop Clin North Am	2003
Hertel [4]	patellar tendon	mini-open	KSSTA	2005
Felmet [6]	patellar tendon	anteromedial	Arch Orthop Trauma Surg	2010
Barie [1]	quadriceps tendon	transtibial	Unfallchirurg (German)	2010

Press-fit fixation of a bone block on the femoral side provides sufficient strength of approximately 400 N when underdrilled by 1 mm and further dilated [11,13]. Further, this fixation type assures a perfect tendon to bone junction on the femoral side, thus helping graft incorporation and early graft function. Using this technique we have not seen any case when the bone block could be pulled out of the femoral tunnel. As no bony defect remains on the femoral side ACL revision surgery may be easier.

On the tibial side, graft fixation is achieved by tying the sutures over a bone bridge, which was shown by Jagodzinsky et al. to provide sufficient strength to allow graft healing [14]. When the tibial tunnel is filled up with bony material the soft tissue graft can be compressed to the tunnel wall therefore helping graft incorporation and providing direct tendon bone healing [15]. Further, bone loss can also be minimized on the tibial side, therefore making revision surgery easier and avoiding the development of large bony defects such as seen with large screws on the tibial side.

The described surgical technique presents several advantages:

· Drilling the femoral bone tunnel through an anteromedial portal allows a more anatomic placement of the ACL replacement graft on the femoral side.

· With press-fit fixation a complete ossification of the femoral bone tunnel can be achieved with a minimal bony defect.

· The technique represents a biological approach, since no artificial fixation devices, i.e. implants, are utilized.

· The tibial tunnel diameter is reduced by filling it with the tibial bone cylinder, therefore decreasing the risk of postoperative bone tunnel enlargement and making revision surgery easier.

· The biomechanical function of hamstring tendons can be preserved which may have a potential advantage for patients with MCL deficiency or patients involved in high demand valgus stress sports such as martial arts (judo, etc.).

Further biomechanical and clinical studies are necessary to evaluate this technique in comparison with others.

## Conclusions

ACL reconstruction with autologous quadriceps tendon graft using press-fit fixation of the bone block on the femoral side and suture post and press-fit bone wedge on the tibial side represents a reproducible and biological surgical technique. This novel technique ensures bone tunnel placement through a low anteromedial portal, thus resulting in a more anatomical placement than using a transtibial approach.

## Competing interests

All authors declare that they do not have a conflict of interests.

## Authors’ contributions

Both authors developed the technique as a modification from the original technique developed by Juergen Huber M.D. Heidelberg, Germany. RA performed the clinical evaluations of all patients, performed the literature review and wrote the manuscript. JH performed all surgeries, performed clinical evaluations of several patients and also was involved in writing the manuscript. All authors have read and approved the final manuscript.

## Pre-publication history

The pre-publication history for this paper can be accessed here:

http://www.biomedcentral.com/1471-2474/13/161/prepub
